# Efficacy of Biological and Physical Enhancement on Targeted Muscle Reinnervation

**DOI:** 10.34133/2022/9759265

**Published:** 2022-09-15

**Authors:** Siyang Zhong, Zijun Zhang, Huan Su, Chenyang Li, Yifeng Lin, Wei Lu, Zhendong Jiang, Lin Yang

**Affiliations:** ^1^Department of Basic Education, Zhuhai Campus of Zunyi Medical University, Zhuhai, China; ^2^School of Resources, Environmental and Chemical Engineering, Nanchang University, China; ^3^CAS Key Laboratory of Human-Machine Intelligence-Synergy Systems, Shenzhen Institutes of Advanced Technology, Chinese Academy of Sciences (CAS), Shenzhen 518055, China

## Abstract

Targeted muscle reinnervation (TMR) is a microsurgical repair technique to reconstruct the anatomical structure between the distal nerve and the muscle stump to provide more myoelectric information to the artificially intelligent prosthesis. Postoperative functional electrical stimulation treatment of the patient's denervated muscle or proximal nerve stump as well as nerve growth factor injection is effective in promoting nerve regeneration and muscle function recovery. In this experiment, we successfully established a TMR rat model and divided Sprague-Dawley (SD) adult male rats into TMR group, TMR + FES group, and TMR + NGF group according to TMR and whether they received FES treatment or NGF injection after surgery, and the recovery effect of rat neuromuscular function was assessed by analyzing EMG signals. Through the experiments, we confirmed that growth factor supplementation and low-frequency electrical stimulation can effectively promote the regeneration of the transplanted nerve as well as significantly enhance the motor function of the target muscle and have a positive effect on the regeneration of the transplanted nerve.

## 1. Introduction

Peripheral nerve injury is caused by various factors such as fracture dislocation, ischemia, and trauma, which often lead to impaired autonomic function, sensory function deprivation, decreased muscle tension, and neuropathic pain seriously affecting the quality of patients' daily lives [1–4]. Currently, many clinical experiments have confirmed that targeted muscle reinnervation (TMR) for patients with peripheral nerve injury can reconstruct the EMG signal lost for amputation by reconstructing the anatomy of the residual peripheral nerve and specific targeted muscle after amputation [5–9].

Despite the therapeutic results of surgery, the postoperative functional recovery of patients with peripheral nerve injury is unideal and falls short of true morphological and functional reconstruction. The difficulty in the treatment of peripheral nerve injury lies in the slow rate of nerve regeneration and function recovery, denervation of skeletal muscle, and the resulting atrophy, degeneration, and dysfunction of target organs innervated by nerves.

The poor functional recovery of patients after TMR is mainly due to the loss of innervated muscles, resulting in varying degrees of atrophy and may even lead to the loss of target organ function, which seriously affects the prognostic repair of TMR surgery [10–12]. When the muscle atrophies to a certain extent, even if the neurological function is restored, the muscle will not contract; so, a certain electrical stimulation needs to be given in time after muscle denervation. Functional electrical stimulation (FES) is a type of neuromuscular electrical stimulation in which a group of muscles is stimulated using a predetermined procedure with a specific intensity of low-frequency pulse current, muscle and joint movements are induced in multiple, or normal voluntary movements are simulated in order to improve or restore the function of the stimulated muscles or muscle groups [13]. It has been shown that FES treatment immediately after TMR may have different effects on the neurological recovery of patients after surgery due to different stimulation parameters, and FES treatment within a certain range can effectively improve the motor function of patients as well as promote the regeneration of sensory nerves [14–16].

In addition, the current basic research shows that NGF can also be applied to peripheral nerve injury repair and is currently the only neurological protein factor that can be applied in clinical practice. Nerve growth factor (NGF) is one of the most essential bioactive molecules in the nervous system, which plays an important role in regulating the growth, development, differentiation, survival of neurons and regeneration, and repair of injured nerves [17, 18]. Zhao et al. [19] experimentally verified that murine nerve growth factor can effectively treat peripheral nerve injury, promote nerve repair and regeneration, and improve the quality of patient's life.

The unresolved issue is how to improve the outcome of peripheral nerve reconstruction after TMR and to accelerate the rate of peripheral nerve regeneration. The results of several studies have shown that low-frequency electrical stimulation can significantly have the ability to stimulate myelin regeneration, upregulate nerve growth factor expression, and accelerate the regeneration rate of injured peripheral nerves [20, 21]. The nerve growth factor can be retrogradely transported in motor, sympathetic, and sensory axons and has the function of promoting the differentiation of nerve cells, which is decisive for the regeneration of injured nerves and the recovery of functions [22]. Therefore, in this experiment, we propose to establish a TMR rat model, treat the rats with low-frequency electrical stimulation and local injection of nerve growth factor, so as to compare the effects of low-frequency electrical stimulation and nerve growth factor on the reconstruction of peripheral nerve injury in rats after TMR, and provide a new method and idea for the clinical treatment of peripheral nerve injury.

## 2. Experimental Procedures

### 2.1. Animal Preparation

Eighteen healthy adult male SD (Sprague-Dawley, SD) rats, aged 7-8 weeks and weighing 230 g-270 g, were selected (experimental animals were provided by the Animal Experiment Center, Shenzhen Institute of Advanced Technology, Chinese Academy of Sciences).

They were maintained in a general animal room with a standard temperature of (22 ± 2)°C, a relative humidity of 40% to 60%, and a 12-hour light and 12-hour dark cycle for protection. Eighteen SD rats were randomly divided into three groups of six rats each: TMR group, TMR + FES group, and TMR + NGF group. All animal experiments were approved by the Animal Research Ethics Committee of the Shenzhen Institute of Advanced Technology, Chinese Academy of Sciences, and all experiments were carried out in accordance with the relevant norms and regulations. Peripheral nerve injury study models were established in all rats. In the TMR group, rats with peripheral nerve injury were treated with targeted muscle nerve function reconstruction to reconstruct the EMG signal lost due to peripheral nerve injury.

### 2.2. Animal Models and Surgical Procedures

The SD rats were placed in the anesthesia machine transparent box, and the inhalation parameter was adjusted to 2% isoflurane. After accomplishing the induction of anesthesia, the rats were removed and fixed on the operating table. The anesthesia was maintained with 2.0% isoflurane by mask until the end of surgery. Each rat's whole forelimb and cranial crest had its hair removed. The surgical site was then cleaned with povidone-iodine and 75% alcohol, and an approximately 2 cm lateral incision was made from the antecubital fossa of the right forearm to the intermuscular furrow along the axilla. The median nerve of the forelimb was investigated after the brachial plexus of the right limb was exposed, and the distal end of the median nerve was divided and anastomosed to the deep pectoral muscle using a 10/0 microsuture adventitial suture method.

**Figure 1:**
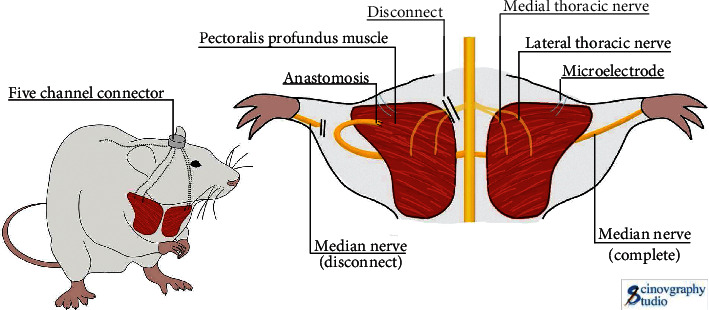
Schematic diagram of TMR surgery in TMR group, TMR + FES group, and TMR + NGF group.

The tenon coating on the Teflon-coated stainless-steel wire was peeled away about 2 mm from the end, the lead was inserted into the deep pectoral muscle, motor EMG signals were obtained, and a reference lead was pulled subcutaneously on the rat's back. Four nails were mounted around the skull “spica,” and the electrode leads were fixed in a circular five-channel connector placed around the skull nail (Figure 1). The connectors were fastened, and the sutures used to repair the incision between the glue and the skull skin were secured with dental cement. To prevent the occurrence of postoperative infection, the surgical region was cleaned, the skin was sutured with 4/0 sutures, and penicillin was administered intramuscularly for three days. The TMR group was as follows: surgery alone and no special treatment after surgery. After separate surgeries on different groups of rats, the rat numbers were remixed, and the cages were randomly assigned according to the random number method to ensure that the rats in each group survived in the same environment and to prevent other confounding factors from influencing the experimental results.

#### 2.2.1. TMR Group

There is a targeted muscle nerve function reconstruction surgery alone in SD rats with peripheral nerve injury, with no special treatment after surgery.

#### 2.2.2. TMR + FES Group

Rats with peripheral nerve injury were treated with low-frequency electrical stimulation therapy instrument after targeted muscle nerve function reconstruction.

#### 2.2.3. TMR + NGF Group

Postoperatively, nerve growth factor was injected into the pectoralis major muscle.

### 2.3. Low-Frequency Electrical Stimulation Procedures

**Figure 2:**
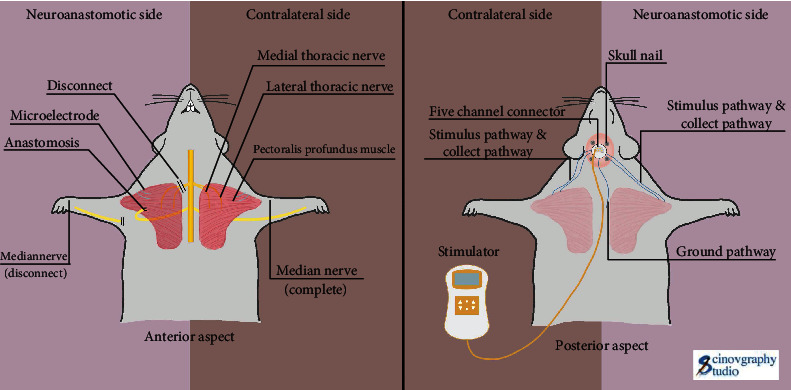
Schematic diagram of electrical stimulation therapy in the TMR + FES group.

The experimental rats in the TMR + FES group were treated with a low-frequency electrical stimulation device on postoperative day 2. Fully implanted bipolar electrodes were implanted in the right deep pectoral muscle of the experimental rats to ensure higher stimulation selectivity with less power (Figure 2). Afterwards, the corresponding 5-channel connectors and leads were connected to the low-frequency electrical stimulation therapeutic instrument. The electrical stimulation setup parameters were set as follows: frequency 20 Hz, pulse width 200 *μ*s, intensity 3-5 mA, current switching ratio 10 s : 10 s, bidirectional symmetric waveform, each treatment time 30 min, once a day, and treatment course 4 weeks.

### 2.4. Nerve Growth Factor Injection

**Figure 3:**
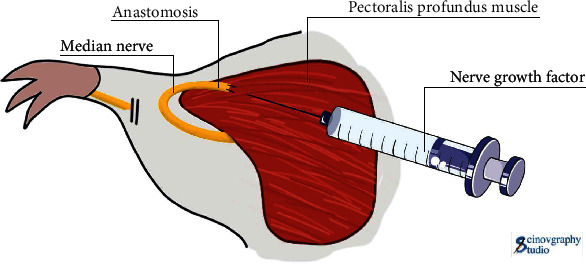
Schematic diagram of injection of NGF.

In the TMR + NGF group, 20 *μ*g of nerve growth factor (Sinobioway Medicine, Co., Ltd., S20060052, Xiamen, China) was injected into the pectoralis major muscle, dissolved in 2 ml of water for injection, and injected intramuscularly, once a day, for 4 weeks as a course of treatment (Figure 3). The research group administered local injection of mouse nerve growth factor 20 *μ*g, dissolved in 2 ml of injection water, and injected locally at the peripheral nerve injury, once a day, for 4 weeks as a course of treatment.

### 2.5. Intramuscular EMG Signal Acquisition

In each group, EMG signals were obtained once a week for four weeks. The five-channel connector was attached to the rat's head connector. A biosignal acquisition system was attached to the electrode output. The corresponding five-channel connector was used to connect to the rat's head connector. The electrode output was connected to a biosignal acquisition system (MedLab-U8C502, Nanjing MedEase Technology Co., Ltd., China). Rats were placed on an animal treadmill for running exercise. Running speed was 9 m/min; exercise was performed for 30 s, the remaining 30 s, and three cycles, which has a total time of 3 min. The EMG signals of the deep pectoral muscles on both sides were also collected. These were the amplifier settings for the Med-lab-U/8 C502 biological signal acquisition and processing system: magnification 1000, upper limit 1000 Hz, time constant 5 s, 50 Hz notch (off), high-pass filtering 10 Hz, and sampling interval 500 *μ*s.

### 2.6. Analysis of Intramuscular Myoelectric Signals

Signal recordings include EMG signals during contraction of reinnervated muscle activity and signals during muscle activity, such as interference, noise, and motion artifacts. In order to extract clear signals for accurate evaluation of pectoralis major, we need to overcome other problems of poor frequency resolution in the high frequency band and poor temporal resolution in the low frequency in the experiment. Therefore, we employed a wavelet packet decomposition and reconstruction approach. Wavelet packet analysis is an integral transform that decomposes signals in the frequency band [23]. By varying the time scale, the local details of the signal can be amplified and narrowed. Wavelet analysis has the characteristics of realizing multiresolution transformation of signals. The following is the wavelet packet decomposition and reconstruction algorithm:

(1) $$\begin{array}{c} WT\left(a,\tau \right)=\frac{1}{\sqrt{a}}\int_{-\infty }^{\infty }f\left(t\right)*\Psi \left(\frac{t-\tau }{a}\right)dt,\\ \phi \left(t\right)=\sqrt{2}\underset{k}{\sum }h_{ok}\phi \left(2t-k\right),\\ \psi \left(t\right)=\sqrt{2}\underset{k}{\sum }h_{1k}\phi \left(2t-k\right). \end{array}$$

In the Daubechies wavelet with *N* = 2, the structure of the db2 wavelet is simple and similar to that of the EMG signal. Therefore, the EMG signal collected after TMR surgery was decomposed using the db2 wavelet basis function with three layers of wavelet packets. S1-S8 represent the eight wavelet packet coefficients, respectively, and the wavelet packet decomposes the corresponding band range of each node. The ARV (average rectified value) of an EMG signal is used in EMG signal analysis to reflect the average absolute value across time. This value represents the average muscle activation level during a given time period, which could be used to compare the levels of the various groups of rats in this experiment. It is defined as Equation (2)

(2) $$\begin{array}{c} X_{arv}=\frac{1}{T}\int_{0}^{t}\left|x\left(t\right)\right|dx. \end{array}$$

### 2.7. Statistical Analysis

SPSS 21.0 software was used for the statistical analysis of ARV values. The measurement data were expressed as (*x* ± *s*), and the analysis of variance was used to compare multiple groups, followed by the independent sample *t*-test. The comparison within the group before and after treatment was performed by a paired *t*-test to determine the statistical significance of the same muscle difference at different times. At *P* < 0.05, a significant difference was discovered.

## 3. Results

### 3.1. General Condition of Rats

All rats drank and ate normally, and there were no complications, infections, and ulcers in the surgical wound. During the treadmill exercise, all rats could complete the running exercise, and there was a discordance between the exercise of the surgical side and the control side. EMG signals were successfully acquired from the target muscles of all 18 rats in the three groups by electrodes implanted within the muscle.

### 3.2. Analysis of Active Myoelectric Signals

**Figure 4:**
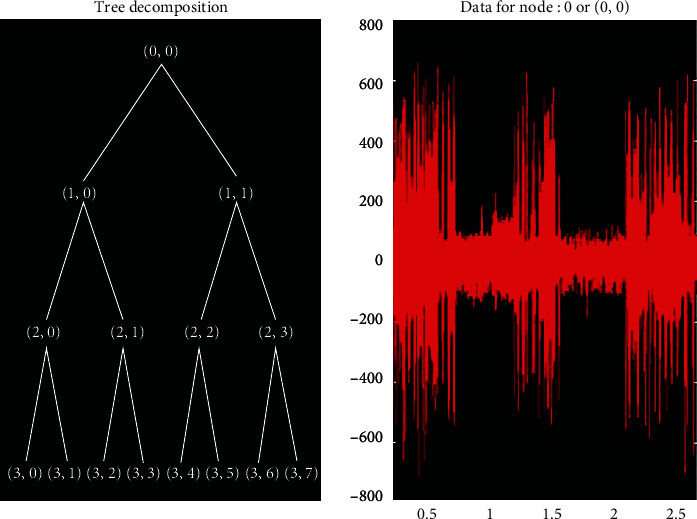
The decomposition corresponding to the three-layer wavelet packet.

After placing the rats on a treadmill with a speed setting of 9 m/min, the electromyographic signals from the target muscle activity were extracted using a dB2 wavelet-based algorithm, and the data were counted and analyzed.  Figure 4 shows the wavelet packet tree, which corresponds to the decomposition of three layers of wavelet packets. The wavelet packet tree can decompose both low-frequency and high-frequency information, enabling better time-frequency localization analysis of signals containing large amounts of medium and high-frequency information. Each node of the wavelet packet tree has its corresponding wavelet packet coefficient, which determines the magnitude of the frequency.

**Figure 5:**
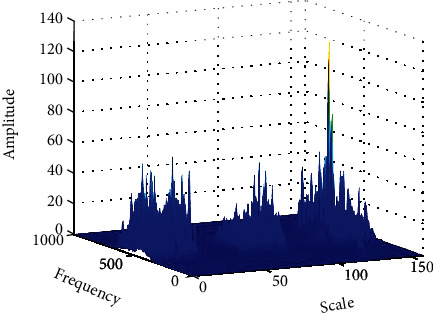
Wavelet time-frequency diagram.

**Figure 6:**
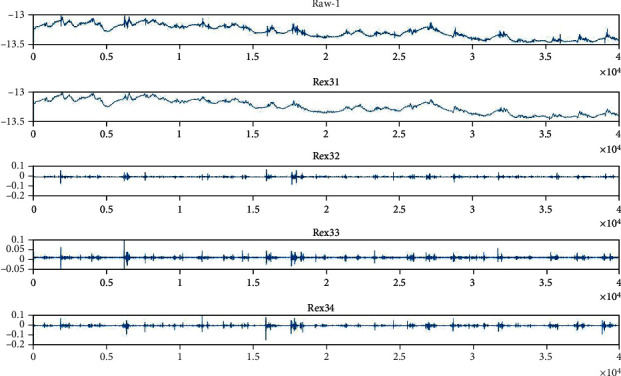
Approximate and detailed coefficients of the wavelet packet triple decomposition reconstruction of the TMR + FES mouse pectoralis major during the first week of running. It can be seen that the noise is mainly in the Rex31 coefficients, raw-1 represents the original EMG signal recorded from channel 1 (right pectoralis major).

Figures 5 and 6 show the approximate and detailed coefficients for the pectoralis major muscle of mice reconstructed from wavelet time-frequency maps and wavelet packet triple decomposition. During the 30 s run, it can be seen that the noise is mainly in the frequency range of Rex1, which may be influenced by the 50 Hz operating frequency.

### 3.3. General Animal Procedures

**Figure 7:**
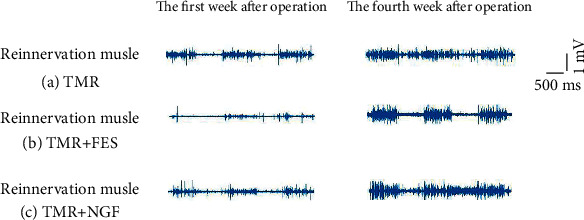
Typical waveform of myoelectric activity.

Figure 7 shows some typical waveforms of the EMG activity within the muscles of rats in the TMR group, rats in the TMR + FES group, and rats in the TMR + NGF group during exercise on a treadmill.

The results showed that the EMG amplitude of the TMR + NGF and TMR + FES group was generally stronger than that of the TMR group at 1 week after surgery, but both were weaker than those of the normal side. The EMG amplitude of the TMR group, the TMR + FES group, and the TMR + NGF group all rose by the 4th postoperative week.

The TMR + FES and TMR + NGF groups were both stronger than the TMR group, but both were still weaker than the normal side.

### 3.4. Analysis of Active Myoelectric Signals

**Figure 8:**
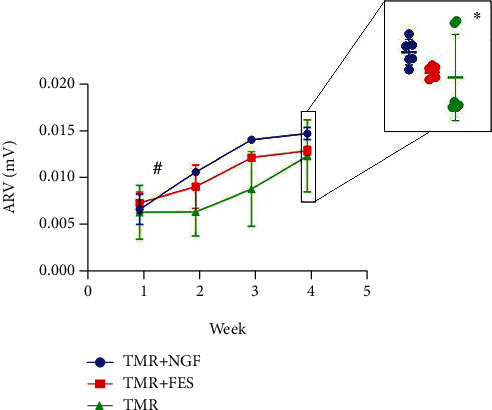
Analysis of active myoelectric signals. #Compared with the fourth week: TMR *P* = 0.001 < 0.05, TMR + FES *P* = 0.000 < 0.05, TMR + NGF *P* = 0.003 < 0.05; ^∗^comparison between the three groups: *P* > 0.05.

The ARV of the EMG signals extracted from the experimental rats was analyzed. A larger value of ARV indicates an increase in EMG amplitude [24]. The ARV of the active EMG signal was analyzed for all rats to reflect the changes in muscle function.  Figure 8 shows the mean muscle activity levels at ARV values for all rats (*n* = 18) in the TMR group, TMR + FES group, and TMR + NGF group. In the TMR + NGF group and the TMR + FES group, there was a significant increase in ARV compared with the TMR group, but the difference was not statistically significant (*P* > 0.05). The ARV in the TMR + NGF group exceeded that in the TMR + FES group at 4 weeks, which may represent a better prognosis than that in the TMR group with time to recovery.

## 4. Discussion

The ARV values of the right pectoralis major muscle in the TMR group, TMR + FES group, and TMR + NGF group increased significantly at week 4 compared with week 1 (*P* < 0.05), in which the right pectoralis major muscle in the TMR + FES group and TMR + NGF group showed more significant growth and better recovery compared with the TMR group at week 4.

We propose a conjecture about this: there is a cumulative effect of both low-frequency electrical stimulation treatment as well as the effect of nerve growth factor on TMR. A cumulative effect is one in which the effect of a single effect over a short period of time may be insignificant and overlooked, but the effect of multiple activities or multiple repetitions of activity superimposed over a long period of time and a large spatial extent is potentially and significantly significant. The increase in right pectoralis major ARV values was not significant in the TMR group at week 4 compared to week 1, while the TMR + FES group showed a significant increase in right pectoralis major ARV values. In addition, considering that the quality of EMG signals recorded through intramuscular electrodes gradually degraded with time, but the right thoracic muscle ARV values in the TMR + FES group as well as in the rats still showed an increasing trend, this indicates that low-frequency electrical stimulation has a positive effect on the recovery of motor function after nerve grafting. The specific process of low-frequency electrical stimulation is to activate local sensory neurons first and then motor neurons through a feedback loop of excitatory synaptic-central connections, thus causing muscle contraction [25, 26]. In contrast, neurotrophic factor mediates neural connections in the peripheral nervous system (PNS). After peripheral nerve injury, NGF binds to nerve growth factor receptors on the surface of Schwann cells and concentrates brain-derived neurotrophic factor (BDNF), which acts together with other substances in the microenvironment associated with axon regeneration, such as laminin and fibronectin, to form a concentration gradient in front of the regenerating axon and guide. In addition, both BDNF and nerve growth factor have neurotropic effect, which can guide the extension direction of regenerated axons to gradually connect with nerve endings and promote the restoration of synaptic connections with the corresponding target organs to facilitate the restoration of nerve conduction function [27]. The mechanism of action of both treatment modalities on the treatment of peripheral nerve injury has a certain time frame of action, leading to a cumulative effect of both low-frequency electrical stimulation treatment and the effect of nerve growth factor to improve the effect of peripheral nerve reconstruction after TMR.

In addition, this may be due to the small sample size of this experiment, which could not eliminate the inability to completely eliminate individual differences between rats, especially the existence of two rats in the TMR group at the fourth week with significantly higher ARV values than rats in the same group, considered as the effect of individual differences on the experimental results. The sensitivity to electrical stimulation treatment in rats of different ages due to the different plasticity of neural functions has an influence on the experimental results. Neuroplasticity is the theoretical basis of neurorehabilitation, which refers to the property of the nervous system to undergo dynamic changes in structure and function to adapt to the changing internal and external environment. Peripheral nerve injury has a stronger regenerative capacity than central nerve injury, and its regenerative capacity is closely related to age, type of injury, and distance from the cell cytosol. The young rats were more sensitive to the electrical stimulation and the upregulation of the nerve growth factor level, and the treatment had a stronger effect on the promotion of axon regeneration and the recovery of nerve function in the young rats than in the old rats. In addition, there were individual differences in the stimulation effect on nerve fibers innervating the antagonist muscle near the electrode because the stimulation current was affected by the hydration of the stimulation site, body hair, and the amount of coupling gel applied. The acquired EMG data of the pectoralis major muscle may be interfered by the signal of the underlying pectoralis minor muscle. Also, the nerves of the deep pectoralis muscle group innervating the pectoralis minor muscle may have a partial trophic effect and compensate for the denervated pectoralis major muscle; so, the experimental effect may not be significant.

The design of this study allowed us to evaluate the effects of bioaugmentation and physical enhancement on nerve function reconstruction in rats. Our experimental results showed that the myoelectric amplitude was generally stronger in the NGF + TMR group as well as the FES + TMR group compared to the TMR group, with more pronounced nerve reconstruction and significantly faster nerve conduction in nerve-injured rats, all of which confirm the potential contained in functional electrical stimulation treatment as well as the use of nerve growth factors in peripheral nerve injury repair.

This is consistent with the notion that electrical stimulation greatly promotes the expression of neurotrophins and their receptors on motoneurons [28–30], and that accelerated upregulation of neurotrophins is an important component of the cellular mechanisms by which brief electrical stimulation promotes axonal regeneration and target nerve regeneration [31]. Short-term electrical stimulation significantly improves the effectiveness of axonal growth at the surgical site of the injured proximal peripheral nerve as well as the distal nerve stump. Compared with transient electrical stimulation, the high-frequency functional electrical stimulation we selected in this study can more effectively preserve the contractile function of type II muscle fibers, maintain their contractility, effectively prevent muscle atrophy, and significantly improve muscle atrophy caused by fast muscle fiber denervation after nerve injury in rats.

Functional electrical stimulation has been favored by researchers in the field of peripheral nerve repair due to its advantages of easy of use, less side effects, and has become a commonly used adjuvant therapy for clinical peripheral nerve repair. Nerve growth factor has the possibility of clinical application in the field of nerve reconstruction because of its advantages of protecting effector neurons and promoting nerve fibers. When studying the therapeutic effect of low frequency electrical stimulation and nerve growth factor on nerve reconstruction, the acquisition, analysis, and statistics of intramuscular EMG signals in rats are extremely important. In this experiment, we chose wavelet transform to analyze the collected EMG signals, which allows us to decompose various intertwined mixed signals composed of different frequencies into signals in different frequency bands and detect many signal characteristics ignored by other analysis methods.

However, it is worth noting that although the experimental results have confirmed that the application of electrical stimulation and nerve growth factor has a significant effect on neurological reconstruction, the study of electrical stimulation and nerve growth factor in peripheral nerve repair is still in its infancy, and there are still many problems that need to be further solved. There are many factors that affect the therapeutic effect of FES, including the time, intensity, frequency, pulse width, duration of electrical stimulation, and the position of electrode placement. In peripheral nerve injury, NGF can repair damaged nerves to varying degrees by promoting the proliferation of Schwann cells and promoting the regeneration of nerves. However, there are still many problems with it. For example, the route of NGF administration and its unique protein spatial structure and its relationship with its activity need to be considered when using nerve growth factor for nerve repair therapeutic effects.

In future studies, in order to address the more limited problem of NGF or FES single action, we will look at the therapeutic effect of FES combined with NGF on neurological reconstruction, to explore the related factors affecting the effect of FES combined with NGF by comparison, and attempt to specify a more applicable rehabilitation program.

## 5. Conclusions

In this experiment, we successfully established a TMR rat model and used intramuscularly implanted electrodes to collect EMG signals from the surgical site of rats and conducted noise reduction and analysis of the acquired EMG signals by wavelet transform to explore the recovery of neuromuscular function in rats with peripheral nerve injury. According to the changes in EMG signals, the transplanted median nerve was successfully regenerated and redistributed in the pectoralis major muscle. We confirmed that the use of low-frequency electrical stimulation therapy and the application of growth factors had an effective influence on the regeneration of the transplanted median nerve.

## Data Availability

The data used to support the findings of this study are included within the article. The data used to support the findings of this study are available from the corresponding author upon request.
